# Integrated Phytochemical Analysis Based on UPLC-MS and Network Pharmacology Approaches to Explore the Quality Control Markers for the Quality Assessment of *Trifolium pratense* L.

**DOI:** 10.3390/molecules25173787

**Published:** 2020-08-20

**Authors:** Liyu Luo, Wenya Gao, Yan Zhang, Chang Liu, Guopeng Wang, Hongwei Wu, Wenyuan Gao

**Affiliations:** 1 School of Pharmaceutical Science and Technology, Tianjin University, Tianjin 300072, China; luoliyu@126.com; 2 Institute of Chinese Materia Medica, China Academy of Chinese Medical Sciences, Beijing 100700, China; gaowenya0215@163.com (W.G.); 17862972336@163.com (Y.Z.); liuchangbj2009@126.com (C.L.); 3 School of Chinese Materia Medica, Beijing University of Chinese Medicine, Beijing 100029, China; binglelly@163.com

**Keywords:** red clover, network pharmacology, UPLC-MS/MS, quality control marker

## Abstract

Red clover consists of the overground parts and inflorescence of *Trifolium pratense* L., a leguminous plant belonging to the genus *Trifolium*. It is widely distributed worldwide and has long been used in traditional medicine. In this study, a combination approach using UPLC-MS and network pharmacology was applied to explore the quality control markers for the quality assessments of red clover. Firstly, UPLC-MS was used to identify the compounds in different parts of red clover. Twenty-eight compounds were totally identified. According to the traditional clinical efficacy of red clover, a compound-target-function network was constructed by network pharmacology to discover the main active compounds based on the identified compounds. Nine compounds of chlorogenic acid, daidzin, calycosin-7-*O*-β-d-glucoside, genistin, ononin, daidzein, genistein, formononetin, and biochanin A were filtrated and further confirmed in rat plasma in view of the blood-absorbed components taking effects. Finally, a novel method for simultaneously detecting the nine quality control markers was developed by UPLC-QQQ-MS in an effort to assess the quality of red clover. For all samples, the average contents of the nine compounds measured from high to low consist of formononetin, ononin, biochanin A, genistin, daidzin, calycosin-7-*O*-β-d-glucoside, genistein, daidzein, and chlorogenic acid. The samples from Gansu province showed the best quality in the three producing areas This study provides new strategies to explore the quality control markers and develops a novel method for the quality assessment of red clover.

## 1. Introduction

Red clover consists of the overground parts and inflorescence of *Trifolium pratense* L., a leguminous plant belonging to the genus *Trifolium*. It is widely distributed worldwide. In China, it can be found as a cultivated or wild plant, with its main distribution in the Gansu, Hubei and Shanxi provinces, and Northeast China [1]. Red clover has long been used in traditional medicine for the treatment of fever, diabetes, hepatitis, skin infections, snake bites, hypertension, and other diseases in several Asian countries, including China, India, and Thailand [2,3,4,5]. Modern pharmacological research has revealed that red clover has a variety of pharmacological activities including antiviral [6,7], anticancer [8,9,10], antibacterial [11,12], anti-inflammatory [13], antioxidant [14,15,16,17], and neuroprotective activities [18,19]. The major active components of red clover are considered to be flavones, isoflavones, coumarins, and volatile oil [20,21,22]. The isoflavones in red clover also have estrogen-like effects, so they are used to prevent osteoporosis [23,24], improve the symptoms of menopause [25,26], and heal wounds [27].

It is known that traditional herbal medicine is characterized by comprehensive medical effects with many components. The chemical markers of quality evaluation should reflect the overall efficacy. Therefore, how to select the quality control markers based on the clinical efficacy of herbal medicine is the key to evaluate the quality. As for red clover, although there are some reports about the quality evaluation of red clover using high-performance liquid chromatography (HPLC) [28,29], gas chromatography-mass spectrometry (GC-MS) [30,31], and liquid chromatography-mass spectrometry (LC-MS) [32,33], there are no reports using an integrated phytochemical analysis and network pharmacology approach to explore the quality control markers for the quality assessment of *Trifolium pratense* L.

In this study, UPLC-ESI-Orbitrap MS/MS was firstly used to identify the compounds in different parts of red clover. Through comparison with the reference substance and the fragmentation pattern analysis, a total of 28 main compounds were identified in the different parts, including stem, leaf and flower. Then, according to the traditional clinical efficacy of red clover and the identified compounds, a network pharmacology method was used to discovered the main active compounds and a “compound-target-function” network was constructed. Nine compounds of chlorogenic acid, daidzin, calycosin-7-*O*-β-d-glucoside, genistin, ononin, daidzein, genistein, formononetin, and biochanin A were selected as the potential quality control markers. Generally, traditional herbal medicine is administered orally, so the components those are absorbed into blood were believed to take effects. The nine potential quality control markers were further confirmed in the rat plasma by UPLC-MS. Finally, a novel method for simultaneously detecting the nine quality control markers was developed by UPLC-QQQ-MS and applied to assess the quality of 15 batches of the red clover samples with different origins.

## 2. Results and Discussion

### 2.1. Metabolomics Profiling Analysis of Red Clover by UPLC-ESI-Orbitrap MS/MS

A total of 28 components were identified from different parts of the red clover, including 24 isoflavones and 4 organic acids. Isoflavones include isoflavone aglycones (7 species), isoflavone glycosides (9 species), and isoflavone malonyl glucosides (8 species). Furthermore, the relative contents of the identified compounds in different parts of red clover were compared based on the response value of quasi-molecular ion peak. Their contents are generally low in stems, high in leaves and flowers, and the species are almost consistent. Detailed information including compound name, retention time, detected ion formula and high resolution mass spectrometry data are all listed in Table S1 of the Supplementary Materials.

**Figure 1:**
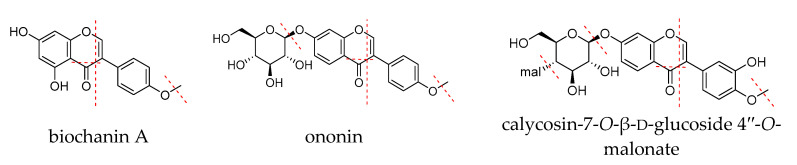
The fragmentation pathways of biochanin A, ononin and calycosin-7-*O*-β-d-glucoside 4”-*O*-malonate.

As the identified components are mainly isoflavones, the characteristics of the fragmentation behaviors for isoflavones are summarized. Isoflavone aglycones usually undergo retro-Diels-Adel (RDA) reaction and loss of small molecule fragments through collision-induced dissociation. In isoflavone glycosides, glycosidic bonds are easily broken and they can lose glycosyl groups to generate aglycon ions. Malonyl glucosides usually lose malonyl groups to generate isoflavone glycosides. For example (as shown in Figure 1), the ion peak [M + H]^+^ of the biochanin A (isoflavone glycoside) in red clover has an *m*/*z* of 285. During collision-induced dissociation, the RDA reaction occurs and fragment ions of *m*/*z* 153 are generated. Small molecule fragments are also lost: A molecule of CH_3_ is lost to generate fragment ion *m/z* 270 due to methoxy bond breakage, and a carbonyl CO is lost to generate fragment ion *m*/*z* 257. Isoflavone glycosides often lose a glucose group during collision-induced dissociation. The formononetin excimer ion peak [M + H]^+^ has an *m*/*z* of 269. This ion loses one molecule of CH_3_ to generate the fragment ion with *m*/*z* 254, and loses one molecule of CO to generate fragment ion *m*/*z* 241, and is fragmented by RDA reaction to generate the fragment ion of *m*/*z* 137. The excimer ion peak [M + H]^+^ of the ononin (isoflavone glycoside) in red clover has a *m*/*z* of 431. Due to the glycosidic bond cleavage, a glucosyl group is lost, generating a fragment ion of *m*/*z* 269 and the other remaining fragment ions are consistent with formononetin. The calycosin-7-*O*-β-d-glucoside 4′′-*O*-malonate is found in red clover and shows a higher response signal in the positive ion mode than in negative ion mode, with an obvious [M + H]^+^ peak. An excimer ion peak with *m*/*z* 533 [M + H]^+^ appears in the first-order mass spectrometry in positive ion mode. The loss of the malonyl glucosyl group produces an ion with *m*/*z* 285, and *m*/*z* 285 loses one molecule of CH_3_ to generate the *m*/*z* 270 fragment ion. The fragment ion *m*/*z* 137 is generated by RDA cleavage.

### 2.2. Quality Control Markers Screening and Validation by Network Pharmacology and Blood-Absorbed Components Analysis

**Figure 2:**
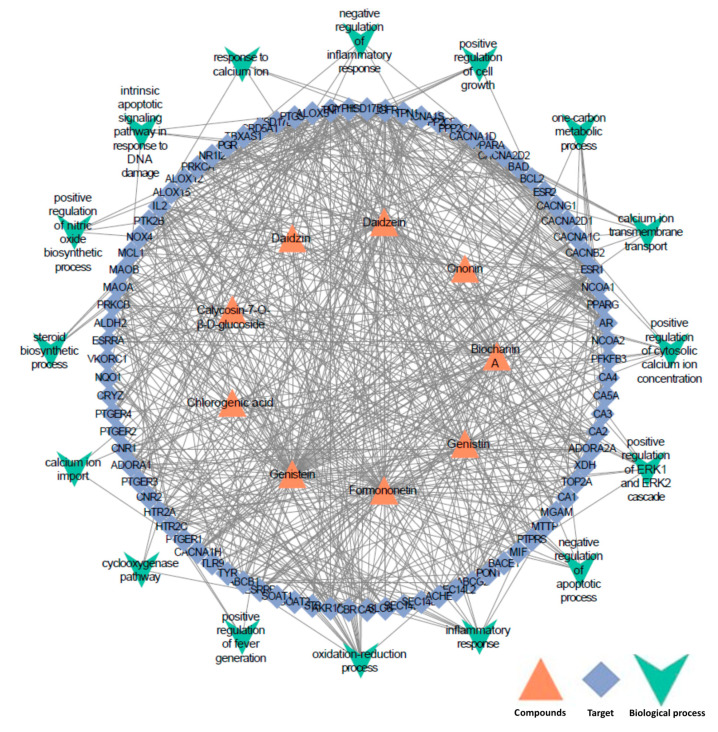
“Component-target-function” network for screening the quality control markers.

A total of 93 high-confidence potential targets of the identified compounds from metabolomics profiling analysis were collected from BATMAN-TCM (http://bionet.ncpsb.org/batman-tcm/) databases (with the score cutoff > 20). Using HPO databases, 294 proteins associated with the clinical effects of red clover were collected. Based on the above data, with the use of the STRING database for the retrieval of protein–protein interactions (PPI score > 0.8), a total of 86 targets had high interaction associations and were finally reserved. These reserved proteins were further imported into the DAVID database (https://david.ncifcrf.gov/) for GO enrichment analysis. One of the enrichment analyses with a Benjamin *p*-value less than 0.05 was selected. Finally, the “component-target-function” network was visualized as shown in Figure 2 using Cytoscape 3.7.1 software. In Figure 2, there are a total of nine compounds (including chlorogenic acid, daidzin, calycosin-7-*O*-β-d-glucoside, genistin, ononin, daidzein, genistein, formononetin, and biochanin A), 86 proteins and 16 pathways.

Of the 16 key pathways, the three pathways of positive regulation of ERK1 and ERK2 cascade (GO: 0070374), inflammatory response (GO: 0006954), and negative regulation of inflammatory (GO: 0050728) response are related to anti-inflammatory effects, which was one of potential mechanisms for the preventing osteoporosis and healing wounds of red clover [34,35,36]. Positive regulation of cytosolic calcium ion concentration (GO: 0007204), calcium ion transmembrane transport (GO: 0070588), and calcium ion import (GO: 0070509) are all involved in the regulation of airway smooth muscle relaxation, which suggests the molecular mechanism of red clover in relieving cough and asthma [37]. In addition, positive regulation of cell growth (GO: 0030307) and negative regulation of apoptotic process (GO: 0043066) play a role in the treatment of burns and cardiovascular disease [38,39,40]. Oxidation-reduction process (GO: 0055114), one-carbon metabolic process (GO: 0006730), steroid biosynthetic process (GO: 0006694), and cyclooxygenase pathway (GO: 0019371) are related to the estrogen activity and anti-cancer activity which were reported for red clover [41,42,43]. The above results reveal the potential mechanisms of the filtered control markers for red clover. Furthermore, the nine potential quality control markers above were extracted by exact mass search in the rat plasma after oral administration of red clover, using the high accuracy of *m/z* determined by UPLC-MS. All the nine compounds were successfully found and conformed by comparing their UPLC retention times and MS data with the authentic commercial standards. Detailed information can be seen in the Supplementary Materials.

### 2.3. UPLC-MS Method Validation

#### 2.3.1. Specificity

**Figure 3:**
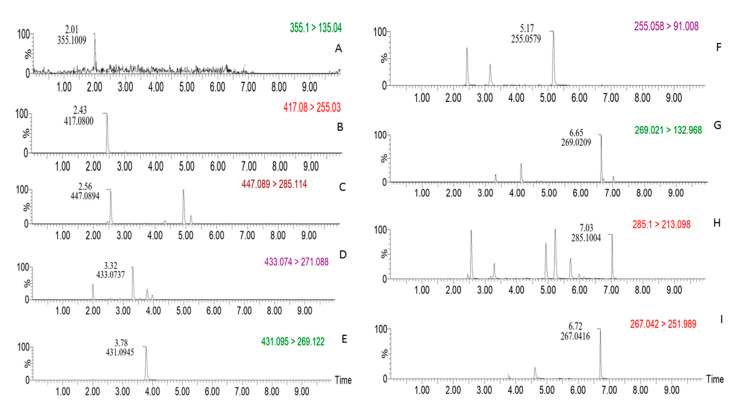
MRM (multiple reaction monitoring) chromatograms of the nine compounds detected in samples. (**A**): Chlorogenic acid; (**B**): daidzin; (**C**): calycosin-7-*O*-β-d-glucoside; (**D**): genistin; (**E**): ononin; (**F**): daidzein; (**G**): genistein; (**H**): biochanin A; (**I**): formononetin.

In this study, a UPLC-MS/MS method was established for simultaneous quantification of the nine compounds with a shortest possible runtime. Typical UPLC chromatograms of multiple reaction monitoring (MRM) for each detected compound are depicted in Figure 3. The specificity of this method was evaluated by comparing the chromatograms of the study samples, reagent blanks and the standards. No other components interfere with the detection of the target compounds. As shown in Figure 3, the nine compounds have ideal chromatographic separations and achieved good peak shape and symmetry factor under the chromatographic and mass spectrometric conditions as described in Section 3.6.2.

#### 2.3.2. Linearity and Limit of Quantitation

Based on the content range of study samples from pre-assessment, a mixed standard stock solution (~15 μg·mL^−1^) was diluted to a series of suitable solution concentrations, which were then analyzed by UPLC-MS/MS. A calibration curve was generated using the peak area of each quantitative ion (Y value) and the corresponding mass concentration (X value, ng/mL), then a linear equation and correlation coefficient were calculated. Good linear correlations for each compounds with r^2^ from 0.9991 to 1.0000 were obtained in the selected ranges. Characteristic parameters for the regression equations and correlation coefficients are given in Table 1. The limits of quantification (LOQ) were determined with the corresponding standard solution at a signal-to-noise (S/N) ratio of 10 for each component, respectively. The results show (Table 1) that LOQs of each compound meet the detection requirements and the method has suitable sensitivity.

#### 2.3.3. Linearity and Limit of Quantitation Precision, Repeatability and Stability

Six consecutive injections of the same sample solution were performed and the relative standard deviation (RSD) values of the peak areas for each compound were between 1.2% and 3.0%, indicating good precision.

To confirm the repeatability, six parallel prepared samples from the same source of red clover were analyzed. The RSD of the contents for the nine compounds detected was 1.2–3.8%, indicating that the method was repeatable.

In order to examine the stability of the sample, a single sample solution stored at room temperature (25 ± 3 °C) was analyzed respectively at 0, 2, 4, 8, 12, and 24 h after preparation. The RSDs of the peak areas for each compound were between 0.8% and 3.9%, indicating that the sample solution is stable within a 24-h period.

#### 2.3.4. Accuracy

The accuracy of the method was determined by spiking a known amount of mixed standards in known red clover samples in triplicate at levels 50% (low), 100% (middle) and 200% (high) of the specified limit. Then the fortified samples were extracted, disposed as described above and analyzed with the procedure. The recoveries were estimated by the formula: Recovery = (amount found − original amount)/amount spiked. The recoveries of nine analytes were calculated and given in Table 1. The recovery of the investigated components ranged from 95.4% to 108.9% and their RSD values were all less than 5.0%, characterizing good reliability and accuracy of the method.

### 2.4. Determination of the Nine Quality Control Markers in 15 Batches of Red Clover

The developed UPLC-MS/MS method was applied to the simultaneous determination of nine quality control markers in collected 15 red clover samples. Furthermore, the total contents of the nine compounds was also calculated. The results are presented in Table 2.

**Figure 4:**
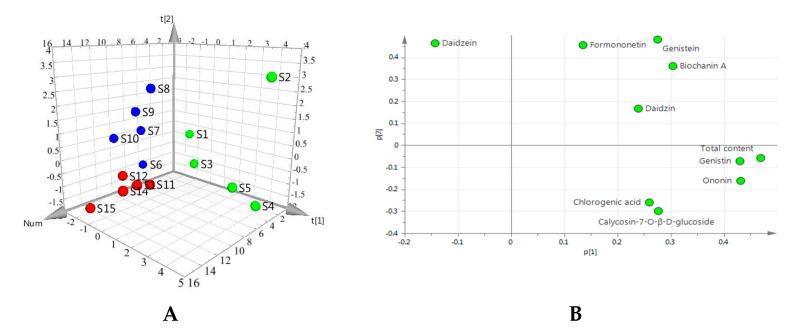
Principal component snalysis. (**A**): Three-dimensional (PCA 3D) score plots using the contents of the nine compounds and the total contents. S1–S5 (green), samples from Gansu; S6–S10 (blue), samples from Hubei; S11–S15 (red), samples from Shanxi. (**B**): Loadings scatter plot.

To investigate the global variations of the quality, we first used principal component analysis (PCA) to analyze all the samples based on the contents of nine components and their total content by SIMCA software (version 13.0). PCA, as an unsupervised pattern recognition method, can reflect the overall quality difference based on all the imported content indexes. As shown in the PCA score 3D plot (Figure 4A), an overview of all samples in the data can be observed and exhibits a clear grouping trend (R^2^X[1] = 0.438; R^2^X[2] = 0.224, Q2 = 0.291) between Gansu group (S1–S5), Hubei group (S6–S10) and Shanxi group (S11–S15). This observation indicates that there is an overall quality difference between the samples from different origins. In the loading scatter plot of PCA (Figure 4B), variables (compounds) situated far away from the origin (on the positive or negative side), especially such as genistin, ononin, total content, dominate the projection. In addition, variables near each other are positively correlated. As shown in Table 2, although the average content of formononetin was the highest in the detected compounds for all the samples, there were no significant differences between the samples from the three producing areas (*p* < 0.05, *t*-test). Except for formononetin, the average contents of all other components and the total content are the highest in the samples from Gansu province. The results indicated that the samples from Gansu province had the best quality in the three producing areas.

For all samples, the average contents of the nine compounds measured from high to low are of formononetin, ononin, biochanin A, genistin, daidzin, calycosin-7-*O*-β-d-glucoside, genistein, daidzein, and chlorogenic acid. In order to discover the potential relationship between the changes in the contents of these compounds, a Spearman correlation analysis was performed using the Software SPSS21.0 (SPSS Inc., Chicago, IL, USA). The Spearman correlation coefficient is the most commonly used measure of monotone association and it is usually suggested for non-normally distributed data. The closer the absolute value of the correlation coefficient (r) is to 1, the more significant the correlation is. Generally, if the absolute value of the correlation coefficient was more than 0.5, it indicated a reliable positive or negative correlation (*p* < 0.05).

**Figure 5:**
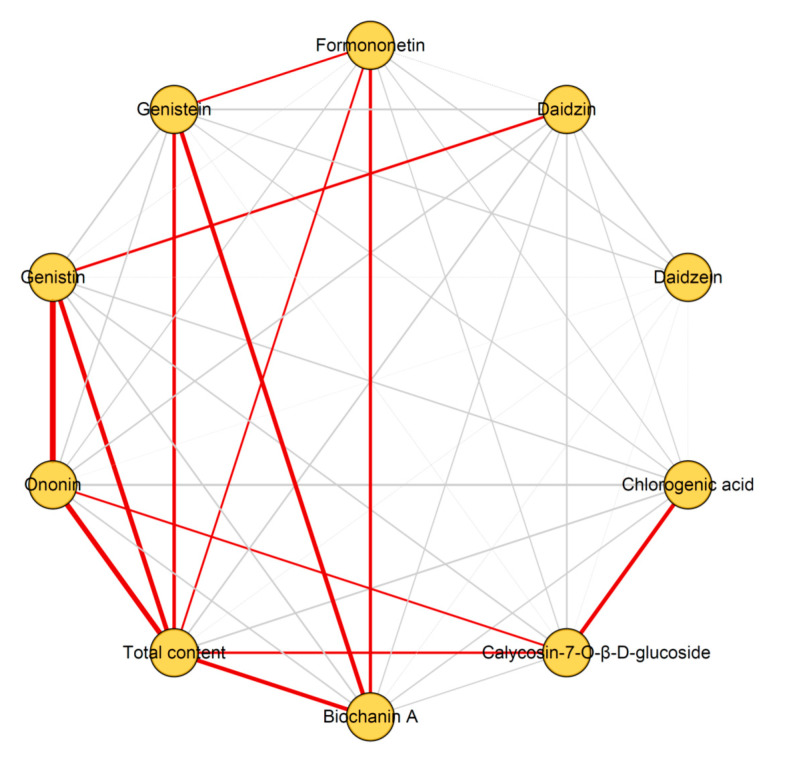
Correlation network for the contents of the nine compounds and the total contents detected in red clover. The significant correlations (|r| > 0.50 and *p* < 0.05) are indicated with red lines. The positive correlations are indicated with solid line. The negative correlations are indicated with dotted lines. The weak correlations (|r| < 0.50) are indicated with grey lines. Thicker lines indicate a stronger correlation. The length of each line has no meaning.

The correlation coefficients between the nine compounds measured are summarized in Table S3 (in the Supplementary Materials). The correlations are depicted visually in Figure 5. Of the nine compounds’ contents and the total contents, the significant correlations (r > 0.5 and *p* < 0.05) are all positive. As shown in Figure 5, the total content with the most significant correlations are total content vs. calycosin-7-*O*-β-d-glucoside, total content vs. genistin, total content vs. ononin, total content vs. genistein, total content vs. formononetin, and total content vs. biochanin A. Secondly, except for chlorogenic acid, daidzin, and daidzein, the other compounds all have three significant correlations. These indicated that the results of the quality evaluation based on the compounds that have significant positive correlations are consistent. In addition, these correlations may be useful in providing evidence for or against specific biosynthetic pathways for red clover.

## 3. Materials and Methods

### 3.1. Instruments and Chemicals

An Ultimate 3000 Ultra High Performance Liquid Chromatography (UPLC) system coupled with LTQ-Orbitrap velos pro mass spectrometer was used (Thermo-Fisher, Waltham, MA, USA), as well as an Acquity UPLC I-Class UPLC system coupled with Xevo TQ-S micro triple quadrupole mass spectrometer (Waters, Milford, MA, USA).

The reference compounds of formononetin, ononin, daidzein, daidzin, genistin, genistein, calycosin-7-*O*-β-d-glucoside, biochanin A, and chlorogenic acid (purity > 98% for all) were all purchased from Shanghai Yuanye Biotechnology Co., Ltd. (Shanghai, China). Acetonitrile and formic acid were purchased from Fisher Scientific, San Jose, CA, USA Company (mass spec-grade). Deionized water was prepared by Milli-Q ultrapure water preparation system (Millipore Co., Ltd., Billerica, MA, USA). All other chemicals were purchased from Beijing Chemical Works (Beijing, China, analytical grade).

### 3.2. Red Clover Sample Collection

A total of 15 batches of red clover were collected from three main areas in the Gansu, Hubei, and Shanxi provinces in China. All the collected samples were denoted as follows: Gansu, S1 to S5; Hubei, S6 to S10; and Shanxi, S11 to S15. The samples were identified as the dry aboveground part (inflorescences, leaf and stems) of the legume *Trifolium pratense* L. by professor Wang Jingjuan of the Beijing University of Chinese Medicine. All samples were stored in a dry, constant environment to minimize any changes through degradation, and the voucher specimens were deposited in our laboratory.

### 3.3. Preparation of Reference Solutions

Accurately weigh an appropriate amount of each reference substance (5~10 mg) into a 50 mL volumetric flask, add methanol to dissolve and dilute to the mark, and obtain a single-component reference stock solution. Precisely measure an appropriate amount of each single-component reference stock solution into another 50 mL volumetric flask, dilute with methanol to the mark, and obtain a mixed reference stock solution with a concentration of ~15 μg·mL^−1^. Then, dilute the mixed reference stock solution to a series of mixed reference solutions at different concentrations.

### 3.4. Metabolomics Profiling Analysis by UPLC-ESI-Orbitrap MS/MS

#### 3.4.1. Sample Preparation for Compounds Identification

The red clover was divided into flower, stem, and leaf portions. Then, 1.0 g powder of each part was accurately weighed and combined with 10mL of 70% methanol, soaked for 1 h and ultrasonically extracted for 30 min. The samples were finally filtrated through a 0.22 μm membrane filter prior to the injection into UPLC-MS system.

#### 3.4.2. Chromatographic and Mass Spectrometric Conditions

Instrument: Ultimate 3000 UPLC instrument, LTQ-Orbitrap velos pro mass spectrometer; column: ACQUITY UPLC BEH Shield RP18 (2.1 × 100 mm, 1.7 μm); flow rate: 0.3 mL/min; column temperature: 40 °C; mobile phase: phase A—0.1% formic acid water, phase B—acetonitrile; injection volume: 2 μL; gradient elution procedure: 0–1.0 min, 90–80% A; 1–5.5 min, 80–60% A; 5.5–6 min, 60–20% A; 6–8 min, 20–5% A; 8–8.5 min, 5% A; 8.5–9 min, 5–90% A; 9–10 min, 90–90% A.

The analysis was performed in positive and negative ion modes. Heater temperature: 350 °C; capillary temperature: 350 °C; capillary voltage: 35 V; spray voltage: 3.4 kV; sheath gas (N_2_) flow rate: 35 arb; auxiliary gas (N_2_) flow rate: 10 arb; mass standard calibration using external standards (mass error is less than 5 ppm). The primary mass spectrum was scanned in FT mode (resolution R is 30,000, and the *m*/*z* scan range is from 50 to 1500). The MS^2^ and MS^3^ spectra were obtained using a data-dependent scan. Dynamic ion exclusion mode was used to obtain additional compound information. Data acquisition and analysis were performed using Xcalibur, Metaworks, Mass Frontier 7.0 software (Thermo Fisher Scientific, San Jose, CA, USA).

### 3.5. Quality Control Markers Screening and Validation by Network Pharmacology and Blood-Absorbed Components Analysis

#### 3.5.1. Quality Control Markers Screening by Network Pharmacology

The protocols included the following four main steps. (1) The potential targets of the all identified compounds in metabolomics profiling analysis were collected from BATMAN-TCM (http://bionet.ncpsb.org/batman-tcm/) databases [44]. In BATMAN-TCM, based on structural similarities between the drug molecules, the proteins with a score cutoff greater than 20 were selected as the potential targets. (2) According to clinical effects of red clover, such as preventing osteoporosis, improving the symptoms of menopause, relieving cough, and healing wounds, the potential targets related to diseases were collected by Human Phenotype Ontology (HPO, https://hpo.jax.org/app/). (3) The proteins of the drug molecular targets and the disease targets from the two databases were entered into the Search Tool (STRING database) for the retrieval of protein–protein interactions (PPI) [45]. Only when the PPI score was greater than 0.8 was the interaction confirmed, and the corresponding proteins and the related compounds were reserved. (4) The reserved proteins were imported into the DAVID database (https://david.ncifcrf.gov/) for GO enrichment analysis [46,47]. Then, one of the enrichment analyses with Benjamin p-value less than 0.1 was selected. Finally, the “component-target-function” network was visualized using Cytoscape 3.7.1 software (https://cytoscape.org/). In addition, in view of the blood-absorbed components taking effects, the potential quality control markers screened by network pharmacology were further analyzed and confirmed in rat plasma after oral administration of red clover by UPLC-MS.

#### 3.5.2. Blood-Absorbed Components Analysis for the Validation of Quality Control Markers

60 g of red clover powder was immersed in 600 mL of 70% Methanol for 1 h, then heated and refluxed for 30 min. After filtration, the extract was concentrated under reduced pressure and freeze-dried to obtain 7.8 g residue. Subsequently, the residue was dissolved in deionised water and the final concentration was 250 mg·mL^−1^.

Male Sprague-Dawley rats (weighing 200 ± 20 g) were obtained from SPF (Beijing, China) Biotechnology Co., Ltd. The animals were acclimatized to laboratory conditions for 7 days and then fasted with free access to water for a 12 h period prior to the experiment. The rats were randomly divided into an experiment group (*n* = 6) and a blank group (*n* = 6). The extracted solution of red clover was administrated by oral administration at a single dose of 5 g·kg^−1^. The blood samples were collected from the orbital venous plexus in a heparinized tube at 0.5, 1, 2, 3, 4, and 8 h after administration. The collected samples were then centrifuged at 4000 rpm for 15 min at 4 °C. The supernatant was removed and then mixed to obtain a pooled plasma. The blank plasma was collected in the same way. All experiments were performed in compliance with related laws and institutional guidelines, and the institutional committees approved the experiments.

A volume of 100 μL of plasma sample was immediately treated with 400 μL acetonitrile and then was vortexed for 3 min to precipitate plasma proteins. After centrifuging at 12,000 rpm for 15 min at 4 °C, the supernatants were evaporated to dryness under nitrogen gas at room temperature. The residue was reconstituted in 100 μL methanol and centrifuged at 12,000 rpm for 15 min at 4 °C, followed by injection of 5 μL into the analysis system. The instrument, chromatographic and mass spectrometric conditions were all the same with “3.4. Metabolomics Profiling Analysis by UPLC-ESI-Orbitrap MS/MS”.

### 3.6. Development and Validation of UPLC-MS/MS Method for Quality Control of Red Clover

#### 3.6.1. Sample Preparation for Quality Evaluation

A total of 1.0 g powder of dried red clover was accurately weighed and placed into a 100 mL vial, with precisely 20 mL of 50% methanol added. The vial was weighed and recorded. Then, the sealed vial was extracted for 30 min by ultrasonication at room temperature. After cooling, 50% methanol was added into the vial to make up to the initial weight. Then, 1 mL of supernatant fluid was diluted into a 25 mL volumetric flask and 50% methanol was used to compensate the rest volume. Samples were finally filtrated through a 0.22 μm membrane filter prior to injection into the UPLC-MS/MS system.

#### 3.6.2. Chromatographic and Mass Spectrometric Conditions

The UPLC-MS/MS system consisted of an Acquity UPLC I-Class UPLC instrument (Waters, Milford, MA, USA) and Xevo TQ-S micro triple-quadrupole mass spectrometer. The separations were achieved on Agilent Extend C18 column (3 mm × 150 mm, 3.5 µm) and the column temperature was 25 °C. The mobile phase consisted of acetonitrile (A) and 0.1% formic acid solution (B), with the gradient elution of 0–23 min, 15–47% A; 23–25 min, 47–80% A; 25–26 min, 80–95% A; 26–30min, 95–95% A. The flow rate was 0.40 mL/min.

Quantification was performed using multiple reaction monitoring (MRM) based on positive and negative ion scanning. The precursor product ion transition and collision energies are listed in Table 3. The other parameters of the mass spectrometer were as follows: electrospray ionization source (ESI source); ion source temperature, 150 °C; capillary voltage, 0.5 kV; desolvating gas, N_2_, flow rate, 1000 L/h; desolvating temperature, 500 °C.

#### 3.6.3. Methodology Validation

The method was validated for specificity, precision, linearity, limit of quantification (LOQ), stability, repeatability and accuracy. The specificity of this method was evaluated by comparing the chromatograms of the study samples, reagent blanks and the standards. Linearity test solutions were prepared by diluting the mixed standards stock solution. The curve of regression between peak areas and concentration were calculated for the nine compounds. The LOQs for the nine compounds were estimated by injecting a series of dilute solutions at known concentration. Precision was determined by analyzing the same sample solution six times within one day. The sample stability was determined by analyzing a single sample solution that was stored at room temperature for 0, 2, 4, 8, 12, and 24 h. Repeatability was determined by analyzing six separate samples from the same source. The accuracy of the assay method was evaluated with the recovery of the standards from samples. Three different quantities (low, medium, high) of the authentic standards were added to the known real sample. The mixtures were extracted as described in Section 3.6.1 and were analyzed using the developed UPLC-MS/MS method.

## 4. Conclusions

In this study, integrated phytochemical analysis and network pharmacology approaches were used to explore the quality control markers for the quality assessment of red clover. Firstly, UPLC-ESI-Orbitrap MS/MS was used to identify the compounds in different parts of red clover. A total of 28 compounds were indeed identified in stem, leaf and flower. The relative contents of the identified compounds are generally low in stems, high in leaves and flowers, and the species are almost consistent. Then, based on the traditional clinical efficacy of red clover and the identified compounds, a “compound-target-function” network was constructed using a network pharmacology method. Nine compounds of chlorogenic acid, daidzin, calycosin-7-*O*-β-d-glucoside, genistin, ononin, daidzein, genistein, formononetin, and biochanin A were selected as the potential quality control markers. Furthermore, the nine potential quality control markers were confirmed in the rat plasma by UPLC-MS. Finally, a novel method for simultaneously detecting the nine quality control markers was developed by UPLC-QQQ-MS and applied to assess the quality of 15 batches of the red clover samples with different origins. This study provides new strategies to explore the quality control markers and developed a novel method for the quality assessment of *Trifolium pratense* L.

## Figures and Tables

**Table 1:** Results of the method validation for the nine compounds detected in red clover.

Compound Name	Linear Range (ng/mL)	*R* ^2^	Limit of Quantitation (ng/mL)	Reproducibility RSD (%) (*n* = 6)	Average Recovery Rate (%)		
					Low a (*n* = 3)	Middle (*n* = 3)	High (*n* = 3)
Chlorogenic acid	20.97~419.42	0.9991	20.97	1.8	103.4	104.2	105.2
Daidzin	1.45~289.14	0.9995	0.014	2.5	96.5	105.2	97.8
Calycosin-7-*O*-β-d-glucoside	1.28~255.43	0.9999	0.013	2.8	96.6	103.5	103.5
Genistin	1.20~239.08	0.9997	0.24	3.8	97.8	101.4	97.3
Ononin	1.66~165.58	0.9998	0.017	1.5	100.4	104.1	97.7
Daidzein	1.51~151.16	0.9992	0.30	2.5	103.7	104.3	100.9
Genistein	12.56~627.98	0.9993	12.56	2.1	108.9	101.3	97.0
Formononetin	1.17~233.16	0.9997	0.50	1.2	102.5	95.4	97.5
Biochanin A	1.77~884.35	0.9997	0.71	2.2	104.7	103.4	97.5

**Table 2:** The contents of the 9 quality control markers in 15 batches of red clover.

Sample Number	Content of Compounds in Red Clover Extract (ng/mL)										Location
	Chlorogenic Acid	Daidzin	Calycosin-7-*O*-β-d-glucoside	Genistin	Ononin	Daidzein	Genistein	Formononetin	Biochanin A	Total Content	
S1	85	7361	3450	5175	33647	5662	3933	35193	11729	106235	Gansu
S2	1717	18395	571	28649	72213	4678	9323	48149	40433	224128	
S3	5710	2880	2812	7970	35019	3146	5696	34597	19793	117623	
S4	7695	11353	29628	18027	72187	3562	4823	50069	28870	226214	
S5	1291	7952	8447	24379	80374	2342	4072	36096	26756	191709	
Average (S1–S5)	3300	9588	8982	16840	58688	3878	5569	40821	25516	173182	
S6	397	5767	2202	3790	14945	4058	2233	22354	4359	60105	Hubei
S7	739	12847	3094	3065	16570	6484	3280	44849	5792	96720	
S8	872	8382	2674	6294	18462	6727	7860	57704	35219	144194	
S9	217	2834	1430	2326	16204	5783	6203	75028	45784	155809	
S10	209	7003	1650	3152	11080	5944	4183	36855	8209	78285	
Average (S6–S10)	487	7367	2210	3725	15452	5799	4752	47358	19873	107023	
S11	4023	3955	5301	9726	39039	2501	4873	46977	34518	150913	Shanxi
S12	-	5457	3254	6584	33063	3033	3734	43176	23020	121321	
S13	-	4155	5628	8145	60609	3268	5434	74243	20612	182094	
S14	-	4701	5162	12056	47835	2475	4997	41490	19992	138708	
S15	1345	2142	2778	6023	24024	1854	3152	33998	15880	91196	
Average (S11–S15)	2684	4082	4425	8507	40914	2626	4438	47977	22804	136846	
Average (S1–S15)	2025	7012	5205	9691	38351	4101	4920	45385	22731	139017	

Note: “-”: indicates not detected.

**Table 3:** Mass spectrometry parameters of the nine detected compounds.

Compound Name	ESI	Molecular Formula	Parent Ion (*m/z*)	Daughter Ion (*m/z*)	Cone Voltage (V)	Collision Energy (eV)
chlorogenic acid	+	C_16_H_18_O_9_	355.10	135.04	30	40
daidzin	+	C_21_H_20_O_9_	417.08	255.03	48	12
calycosin-7-*O*-β-d-glucoside	+	C_22_H_22_O_10_	447.09	285.11	52	16
genistin	+	C_21_H_20_O_10_	433.07	271.09	24	16
ononin	+	C_22_H_22_O_9_	431.09	269.12	50	14
daidzein	+	C_15_H_10_O_4_	255.06	91.01	82	18
genistein	−	C_15_H_10_O_5_	269.02	132.97	74	28
formononetin	−	C_16_H_12_O_4_	267.04	251.99	58	16
biochanin A	+	C_16_H_12_O_5_	285.10	213.00	74	34

## Supplementary Materials

The Supplementary Materials are available online.
