# Supplementary material for: Integrated Phytochemical Analysis Based on UPLC-MS and Network Pharmacology Approaches to Explore the Quality Control Markers for the Quality Assessment of Trifolium pratense L
Source: Molecules. 2020 Aug 20;25(17):3787. doi: 10.3390/molecules25173787 (PMC7504318; doi:10.3390/molecules25173787)
Supplement: Supplementary file 1 [file molecules-25-03787-s001.pdf]

Supplementary Materials

1 Metabolomics profiling analysis of red clover

Table S1. Compound identification of red clover in positive and negative ion modes.

No.	RT min	Ion Form	Ion Formula	MS[1] Measured *m/z*	MS[1] Predicted *m/z*	MS[1] Err ppm	MS[2]	MS[3]	Identification	Stem	Leaf	Flower
1	1.43	$[M-H]^-$	$C_6H_{11}O_7$	195.0505	195.0499	2.82			Gluconic acid	++	++	+++
2	4.46	$[M-H]^-$	$C_{13}H_{11}O_8$	295.0450	295.04484	0.67	**179.03506($C_9H_7O_4$);** 133.01451($C_4H_5O_5$); 115.00400($C_4H_3O_4$);	[295-179]135.04524($C_8H_7O_2$);	Benzoylcitronensaure	++	+++	+++
3	5.42	$[M+H]^+$	$C_{16}H_{19}O_9$	355.1028	355.1024	1.21			chlorogenic acid	+	+++	+++
4	6.60	$[M+H]^+$	$C_{21}H_{21}O_{10}$	433.1114	433.1129	−3.54	271.06067($C_{15}H_{11}O_5$);	[433-271]253.04956($C_{15}H_9O_4$); 243.06529($C_{14}H_{11}O_4$); 215.07042($C_{13}H_{11}O_3$); **153.01839($C_7H_5O_4$);** 149.02335($C_8H_5O_3$);	Genistein-glucoside	+++	+++	+++
5	6.71	$[M+H]^+$	$C_{21}H_{21}O_9$	417.1170	417.1180	−2.34	239.07021($C_{15}H_{11}O_3$)		daidzin	+	++	+++
6	7.20	$[M+H]^+$	$C_{22}H_{23}O_{10}$	447.1277	447.1286	−1.91	269.08072($C_{16}H_{13}O_4$)		calycosin-7-*O*-β-D-glucoside	+++	+++	+++

7	8.03	$[M+H]^+$	$C_{25}H_{25}O_{13}$	533.1271	533.1290	−3.54	285.07642($C_{16}H_{13}O_5$);	[533-285]270.05258($C_{15}H_{10}O_5$); 253.04982($C_{15}H_9O_4$); 225.05478($C_{14}H_9O_3$); 137.02333($C_7H_5O_3$);	Calycosin-7-*O*-β-D-glucoside 4″-*O*-malonate	+++	+++	+++
8	8.95	$[M+H]^+$	$C_{21}H_{21}O_{10}$	433.1120	433.1129	−2.11	255.06516($C_{15}H_{11}O_4$)		genistin	++	+++	+++
9	9.31	$[M+H]^+$	$C_{24}H_{23}O_{13}$	519.1116	519.1133	−3.25	433.11377($C_{21}H_{21}O_{10}$); **271.06073($C_{15}H_{11}O_5$);**	[519-271]253.04958($C_{15}H_9O_4$,); 243.06526($C_{14}H_{11}O_4$); **215.07045($C_{13}H_{11}O_3$);** 153.01833($C_7H_5O_4$); 149.02333($C_8H_5O_3$);	Genistein-glucoside malonate	-	+++	+++
10	9.64	$[M+H]^+$	$C_{22}H_{21}O_{10}$	445.1112	445.1129	−3.98	**283.06073($C_{16}H_{11}O_5$);** 253.04929($C_{15}H_9O_4$); 225.05496($C_{14}H_9O_3$);		Pseudobaptigenin 7-*O*-β-D-glucoside	+++	+++	+++
11	10.19	$[M+H]^+$	$C_{22}H_{23}O_9$	431.1323	431.1336	−3.18	**269.08151($C_{16}H_{13}O_4$);** 254.05960($C_{15}H_{10}O_4$);	[431-269]**254.05771($C_{15}H_{10}O_4$);** 213.09125($C_{14}H_{13}O_2$); 137.02341($C_7H_5O_3$); 136.01564($C_7H_4O_3$);	Ononin	+	+++	+++
12	10.5	$[M+H]^+$	$C_{25}H_{25}O_{14}$	549.1225	549.1239	−2.52	463.12491($C_{22}H_{23}O_{11}$); **301.07153($C_{16}H_{13}O_6$);**	**[549-301]286.04773**($C_{15}H_{10}O_6$); 269.04495($C_{15}H_9O_5$); 245.08113($C_{14}H_{13}O_4$); 241.04996($C_{14}H_9O_4$); 153.01846($C_7H_5O_4$); 149.02351($C_8H_5O_3$);	Trihydroxy-methoxyflavone-glucoside-malonate	-	+++	+++
13	11.02	$[M-H]^-$	$C_7H_5O_3$	137.02403	137.0233	5.18			Salicylic acid	+++	+++	+++

14	11.47	[M + H]+	$C_{15}H_{11}O_4$	255.0640	255.0652	−4.65	237.05475($C_{15}H_9O_3$); 227.07045($C_{14}H_{11}O_3$); 209.05980($C_{14}H_9O_2$); **199.07558($C_{13}H_{11}O_2$);** 181.06471($C_{13}H_9O$); 145.02844($C_9H_5O_2$); 137.02345($C_7H_5O_3$);		Daidzein	+	+++	+++
15	12.16	[M + H]+	$C_{25}H_{23}O_{13}$	531.1116	531.1133	−3.29	283.06085($C_{16}H_{11}O_5$);	**[531-283]253.04999($C_{15}H_9O_4$);** 225.05502($C_{14}H_9O_3$); 197.05977($C_{13}H_9O_2$);	Pseudobaptigenin 7-*O*-β-glucoside 6″-*O*-malonate	+++	+++	++
16	12.28	[M + H]+	$C_{22}H_{21}O_{11}$	461.1068	461.1078	−2.36	299.05402($C_{16}H_{11}O_6$);	**[461-299]269.04468(**$C_{15}H_9O_5$**)**; 241.04973($C_{14}H_9O_4$); 225.05496($C_{14}H_9O_3$); 181.01332($C_8H_5O_5$);	Deirisin -4′-*O*-β-D-glucoside Spinonin	-	+++	+++
17	12.65	[M + H]+	$C_{25}H_{25}O_{12}$	517.1320	517.1340	−3.93	269.08173($C_{16}H_{13}O_4$);	**[517-269]254.05789($C_{15}H_{10}O_4$);** 237.05495($C_{15}H_9O_3$); 226.06264($C_{14}H_{10}O_3$); 213.09138($C_{14}H_{13}O_2$); 137.02356($C_7H_5O_3$);	Formononetin 7-*O*-β-D-glucoside -*O*-malonate	+	+++	+++
18	13.76	[M + H]+	$C_{22}H_{23}O_{10}$	447.1271	447.1286	−3.27	285.07651($C_{16}H_{13}O_5$);	[447-285]**270.05261($C_{15}H_{10}O_5$)**; 229.08620($C_{14}H_{13}O_3$); 152.01059($C_7H_4O_4$);	Biochanin A-β-D-glucoside	+++	+++	+++
19	14.3	[M + H]+	$C_{22}H_{23}O_{10}$	447.1270	447.1286	−3.56	285.07626($C_{16}H_{13}O_5$);	[447-285]257.08096($C_{15}H_{13}O_4$); 229.08606($C_{14}H_{13}O_3$); 167.03397($C_8H_7O_4$); 175.03906($C_{10}H_7O_3$);	Prunetin -4′-*O*-β-D-glucoside	+++	+++	+++

20	14.61	$[M+H]^+$	$C_{25}H_{23}O_{14}$	547.1063	547.1082	−3.49	**299.05591($C_{16}H_{11}O_6$);**	151.03908($C_8H_7O_3$); **123.04417($C_7H_7O_2$);** **[547-299]269.04462**($C_{15}H_9O_5$); 243.06537($C_{14}H_{11}O_4$); 241.04973($C_{14}H_9O_4$); 225.05486($C_{14}H_9O_3$); 181.01329($C_8H_5O_5$);	Deirisin -4′-*O*-β-D-glucoside -*O*-malonate	+	+++	+++
21	15.83	$[M+H]^+$	$C_{15}H_{11}O_5$	271.0592	271.0601	−3.32	253.04976(C15H9O4); 243.06548(C14H11O4); 215.07062($C_{13}H_{11}O_3$); **153.01849($C_7H_5O_4$);**		Genistein	-	+++	+++
22	15.89	$[M+H]^+$	$C_{25}H_{25}O_{13}$	533.1265	533.1290	-4.70	447.13031($C_{22}H_{23}O_{10}$); **285.07681($C_{16}H_{13}O_5$);**	[533-285]270.05258($C_{15}H_{10}O_5$); 253.04982($C_{15}H_9O_4$); **229.08633($C_{14}H_{13}O_3$);** 179.03415($C_9H_7O_4$); 149.02351($C_8H_5O_3$); 123.04421($C_7H_7O_2$);	Biochanin A-β-D-glucoside-*O*-malonate	+++	+++	+++
23	16.39	$[M+H]^+$	$C_{25}H_{25}O_{13}$	533.12744	533.1290	−2.87	**285.0749($C_{16}H_{13}O_5$);**	152.01060($C_7H_4O_4$); [533-285]270.0509($C_{15}H_{10}O_5$); **229.0849($C_{14}H_{13}O_3$);** 179.0329($C_9H_7O_4$); 167.0331($C_8H_7O_4$);	Biochanin A-β-D-glucoside-*O*-malonate isomer	+++	+++	+++
24	16.97	$[M+H]^+$	$C_{16}H_{13}O_6$	301.0692	301.0707	−4.83	**286.04752($C_{15}H_{10}O_6$)**; 269.04486($C_{15}H_9O_5$); 241.05003($C_{14}H_9O_4$); 153.01851($C_7H_5O_4$);		Pratensein	-	+++	+++

25	18.7	$[M+H]^+$	$C_{16}H_{11}O_5$	283.0588	283.0601	−4.66	**253.04979($C_{15}H_9O_4$);** 225.05496($C_{14}H_9O_3$); 197.05974($C_{13}H_9O_2$); 183.04405($C_{12}H_7O_2$); 169.06487($C_{12}H_9O$);	Pseudobaptigenin	-	+++	+++
26	19.27	$[M+H]^+$	$C_{16}H_{13}O_4$	269.0800	269.0808	−3.25	**254.05817($C_{15}H_{10}O_4$);** 241.08643($C_{15}H_{13}O_3$); 237.05533($C_{15}H_9O_3$); 213.09163($C_{14}H_{13}O_2$); 137.02341($C_7H_5O_3$); 118.04154(C_8H_6O);	Formononetin	+	+++	+++
27	21.93	$[M+H]^+$	$C_{16}H_{11}O_6$	299.0537	299.0550	−4.56	**269.04492($C_{15}H_9O_5$);** 243.06557($C_{14}H_{11}O_4$); 241.04990($C_{14}H_9O_4$); 225.05492($C_{14}H_9O_3$); 181.01347($C_8H_5O_5$);	Irilone	+	+++	+++
28	24.72	$[M+H]^+$	$C_{16}H_{13}O_5$	285.0748	285.0758	−3.23	**270.05292($C_{15}H_{10}O_5$);** 257.08127($C_{15}H_{13}O_4$); 253.05009($C_{15}H_9O_4$); 229.08647($C_{14}H_{13}O_3$); 153.01862($C_7H_5O_4$); 152.01076($C_7H_4O_4$); 149.02357($C_8H_5O_3$); 123.04431($C_7H_7O_2$);	Biochanin A	-	+++	+++

Bold characters: the base peaks in MS^n spectra; -: means not detected. +++: the response value of the sample is 80–100% of the maximum; ++: the response value of the sample is 40–80% of the maximum; +: the response value of the sample is within 40% of the maximum.

2. Blood-absorbed components analysis for the validation of the quality control markers

Generally, traditional herbal medicine is administered orally, so the components that are absorbed into blood take effect. Therefore, the nine potential quality control markers screened by network pharmacology were further confirmed in the plasma by UPLC-MS.

The nine potential quality control markers screened by network pharmacology were extracted by exact mass search in the plasma, using the high accuracy of m/z determined by UPLC-MS. All the nine compounds were successfully found and conformed as shown in Table S1. The identification was confirmed by comparing their UPLC retention times and MS data with the authentic commercial standards.

Table 2. Blood-absorbed components analysis for the validation of the quality control markers.

No.	RT min	Ion Form	molecular Formula	MS^1			Identification
				Measured *m/z*	Predicted *m/z*	Err ppm	
1	10.06	$[M+H]^+$	$C_{22}H_{22}O_9$	431.1329	431.1336	−1.69	ononin
2	11.22	$[M+H]^+$	$C_{15}H_{10}O_4$	255.0638	255.0652	−5.51	daidzein
3	5.22	$[M+H]^+$	$C_{16}H_{18}O_9$	355.1011	355.1024	−3.56	chlorogenic acid
4	6.53	$[M+H]^+$	$C_{21}H_{20}O_9$	417.1162	417.1180	−4.38	daidzin
5	7.01	$[M+H]^+$	$C_{22}H_{22}O_{10}$	447.1266	447.1286	−4.41	calycosin-7-*O*-β-D-glucoside
6	15.51	$[M+H]^+$	$C_{15}H_{10}O_5$	271.0585	271.0601	−5.79	Genistein
7	19.00	$[M+H]^+$	$C_{16}H_{12}O_4$	269.0793	269.0808	−5.78	Formononetin
8	8.75	$[M+H]^+$	$C_{21}H_{20}O_{10}$	433.1109	433.1129	−4.51	genistin
9	24.47	$[M+H]^+$	$C_{16}H_{12}O_5$	285.0738	285.0758	−6.77	Biochanin A

3 The results of correlation analysis for the contents of nine compounds and the total contents

Table S3. The results of correlation analysis

		Chlorogenic Acid	Daidzin	Calycosin-7-*O*-β-D-glucoside	Genistin	Ononin	Daidzein	Genistein	Formononetin	Biochanin A	Total Content
Chlorogenic acid	Pearson correlation	1	0.017	0.735**	0.344	0.497	−0.485	0.122	0.025	0.227	0.486
	P value（bilateral）		0.958	0.006	0.273	0.100	0.110	0.706	0.938	0.478	0.109
	N	12	12	12	12	12	12	12	12	12	12
Daidzin	Pearson correlation	0.017	1	0.191	0.588*	0.379	0.407	0.414	−0.019	0.122	0.439
	p value（bilateral）	0.958		0.495	0.021	0.164	0.132	0.125	0.947	0.665	0.102
	N	12	15	15	15	15	15	15	15	15	15
calycosin-7-O-β-D-glucoside	Pearson correlation	0.735**	0.191	1	0.358	0.534*	−0.242	−0.094	0.082	0.119	0.532*
	p value（bilateral）	0.006	0.495		0.190	0.040	0.384	0.740	0.772	0.673	0.041
	N	12	15	15	15	15	15	15	15	15	15
Genistin	Pearson correlation	0.344	0.588*	0.358	1	0.895**	−0.353	0.464	−0.031	0.446	0.793**
	p value（bilateral）	0.273	0.021	0.190		0.000	0.197	0.081	0.912	0.096	.000
	N	12	15	15	15	15	15	15	15	15	15
Ononin	Pearson correlation	0.497	0.379	0.534*	0.895**	1	−0.494	0.314	0.129	0.368	0.849**

	p value (bilateral)	0.100	0.164	0.040	0.000		0.061	0.254	0.647	0.178	0.000
	N	12	15	15	15	15	15	15	15	15	15
Daidzein	Pearson correlation	−0.485	0.407	−0.242	−0.353	−0.494	1	0.267	0.250	−0.024	−0.186
	p value (bilateral)	0.110	0.132	0.384	0.197	0.061		0.337	0.369	0.933	0.507
	N	12	15	15	15	15	15	15	15	15	15
Genistein	Pearson correlation	0.122	0.414	−0.094	0.464	0.314	0.267	1	0.540*	0.762**	0.637*
	p value (bilateral)	0.706	0.125	0.740	0.081	0.254	0.337		0.038	0.001	0.011
	N	12	15	15	15	15	15	15	15	15	15
Formononetin	Pearson correlation	0.025	−0.019	0.082	−0.031	0.129	0.250	0.540*	1	0.634*	0.534*
	p value (bilateral)	0.938	0.947	0.772	0.912	0.647	0.369	0.038		0.011	0.041
	N	12	15	15	15	15	15	15	15	15	15
Biochanin A	Pearson correlation	0.227	0.122	0.119	0.446	0.368	−0.024	0.762**	0.634*	1	0.735**
	p value (bilateral)	0.478	0.665	0.673	0.096	0.178	0.933	0.001	0.011		0.002
	N	12	15	15	15	15	15	15	15	15	15
Total content	Pearson correlation	0.486	0.439	0.532*	0.793**	0.849**	−0.186	0.637*	0.534*	0.735**	1
	p value (bilateral)	0.109	0.102	0.041	0.000	0.000	0.507	0.011	0.041	0.002	
	N	12	15	15	15	15	15	15	15	15	15

**. p <0.01; *. p < 0.05
